# The Use of Interleukine-1 Inhibitors in Familial Mediterranean Fever Patients: A Narrative Review

**DOI:** 10.3389/fimmu.2020.00971

**Published:** 2020-05-28

**Authors:** Véronique Hentgen, Caroline Vinit, Antoine Fayand, Sophie Georgin-Lavialle

**Affiliations:** ^1^General Pediatric Department, French National Reference Center for Autoinflammatory Diseases (CEREMAIA), Versailles Hospital, Versailles, France; ^2^Internal Medicine Department, French National Reference Center for Autoinflammatory Diseases (CEREMAIA), Tenon Hospital, Sorbonne University, Paris, France

**Keywords:** familial mediterranean fever, anakinra, canakinumab, interleukine-1, colchicine, autoinflammation, amyloidosis

## Abstract

**Purpose:** Familial Mediterranean fever (FMF) is the most common monogenic auto-inflammatory disease characterized by recurrent attacks of fever and serositis. It is associated with mutation in pyrin inflammasome leading to interleukin-1 (IL-1) over secretion. Although colchicine is the first line treatment in FMF, 5–10% of patients are reported in literature as non-responders. Colchicine is not always well-tolerated due either to its direct toxicity or to co-morbidities that preclude the administration of its proper dosage. For these patients an alternative or additional treatment to colchicine is necessary. This literature review reports the published data regarding the use of IL-1 inhibitors in Familial Mediterranean Fever.

**Results:** There is no uniform definition of colchicine resistance, but the different studies of treatment with IL-1 inhibitors provide evidence of IL-1 pathogenic role in colchicine-resistant FMF. IL-1 inhibition is an efficacious option for controlling and preventing flares –at least at the short term- in FMF patients who are insufficiently controlled with colchicine alone. Although canakinumab is the only approved drug in Europe for colchicine resistant FMF treatment, experience with anakinra is also substantial. In the absence of comparative studies both treatments seem to be an equal option for the management of these patients. Overall the safety profile of IL-1 inhibitors seems not different in FMF patients than in the other diseases and can be considered as globally safe. The main side effects are local injection site reactions and infections.

**Conclusion:** IL-1 inhibitors have the potential to improve patient outcome even in FMF patients with co-morbidities or severe complications in whom inflammation control is difficult to achieve with colchicine alone. Nevertheless, current data are limited and further evaluation of long-term efficacy and safety of IL-1 inhibitors are necessary, in order to provide robust evidence in this domain.

## Key Messages

- There is no uniform definition of colchicine resistance. A standardized evaluation of adherence to colchicine treatment is mandatory before considering IL-1 inhibitors in FMF patients
- Although canakinumab is the only drug approved in Europe for the treatment of colchicine resistant FMF, experience with anakinra is more substantial in the literature.
- There are no comparative effectiveness assessment studies for canakinumab vs. anakinra. Both treatments seem to be an option for the management of colchicine resistant or intolerant FMF patients.
- The use of anakinra in pre-attack prodromal period (“on-demand treatment”) may be a reasonable approach for alleviating symptoms of an impending attack in patients with insufficient response to the maximum tolerated dosage of colchicine and low risk of amyloidosis.
- The use of IL1 inhibitors as a maintenance therapy in patients unresponsive or intolerant to colchicine is globally safe and effective.

## Introduction

Familial Mediterranean fever (FMF) is the most frequent monogenic auto-inflammatory disease. FMF is characterized by self-limited episodes fever associated to polyserositis and raised inflammatory markers (1). The disease is mostly seen among particular ethnic groups such as patients with a Middle Eastern ancestry or originating from the Mediterranean basin (2). Autosomal recessive mutations in the *MEFV* gene are responsible for the symptoms in FMF (3, 4). Although its pathogenesis is not fully understood, pyrin is a crucial player in the regulation of innate immunity and FMF-associated missense mutations induce an uncontrolled IL-1 release (5).

Amyloid deposition and the development of end-stage renal disease are the most severe complications of FMF. Since 1972, colchicine is the cornerstone of treatment for FMF patients. To date, only the daily intake of colchicine has proven its effectiveness on the long-term in preventing or improving inflammatory attacks, but also in decreasing the frequency of secondary amyloidosis (6–8).

Nevertheless, cases of unresponsiveness to colchicine have been reported, although this situation remains rare, probably <10 % of FMF patients (9–11). In addition, colchicine treatment is not always well-tolerated due either to direct colchicine toxicity or to co-morbidities that preclude the administration of the proper colchicine dosage. For these patients an alternative or additional treatment to colchicine is necessary. IL-1 inhibitors are the first candidates given the involvement of IL-1 in pathophysiology of the inflammatory attacks. Four biologic drugs blocking IL-1 are currently available. Of them, anakinra, and canakinumab have been approved for clinical use in Europe, whereas the soluble decoy IL-1-receptor, rilonacept, and the human-engineered monoclonal anti-IL-1, gevokizumab, are not authorized in European countries.

However, the precise indications for initiating IL-1 blocking agents in FMF patients are still unclear and poorly codified. Given the cost of these biological agents and their potential risk of side effects (mainly infections), their use needs still to be defined.

The objective of this article is to review the current knowledge about the use of IL-1 inhibitors in FMF, with the aim of defining the indications and the place of these more recent products in the therapeutic arsenal of the disease.

## Methods

### Literature Search Strategy

A literature search on the use of IL-1 inhibitors and FMF was conducted from 1947 until 2019 using the Medline, Embase, and Cochrane databases using the following terms: “anakinra,” “canakinumab,” “IL-1 inhibitor,” “Interleukin 1 Receptor Antagonist Protein,” “IL-1 blockade” and “familial Mediterranean fever.” The terms were combined as both key words and MeSH terms. We excluded articles about rilonacept and gevokizumab, as both agents are not authorized in European countries.

Additional articles were retrieved by checking manually the references of the recovered articles and the “related articles” function on Pub-Med (www.pubmed.gov) were also assessed for possible inclusions. Only articles published in English or French before September 2019 have been included to this review.

### Data Assessment

All four coauthors read and approved the retrieved articles. We extracted data of the selected articles using predefined scoring forms and classification tables that enabled us to analyze the published data in five different domains: 1/ indications for IL-1 inhibitors in FMF, 2/ efficacy of IL-1 inhibitors in FMF, 3/ comparison of anakinra vs. canakinumab in FMF, 4/ comparison of maintenance vs. on-demand treatment in FMF and 5/ safety of IL-1 inhibitors in FMF.

## Results

Sixty one studies or case reports or series concerning 811 patients were identified: 30 case reports or case series with 5 or less patients, 29 case series or open studies with more than 5 patients and 2 randomized studies. Five hundred and seventy one patients (70.4%) originated from the Middle East, 140 (17.2%) from Europe, 99 (12.2%) from international studies or registries and 1 patient from the USA. The retrieved articles are detailed in Table 1.

**Table 1:** References of the articles described in the literature review.

**Bibliography number**	**First author**	**Number of FMF patients**	**Number of patients treated with Anakinra**	**Number of patients treated with Canakinumab**	**Number of patients treated with both**	**Median age**	**Description of patients with AA amyloidosis**
Chae et al. (12)	Chae	1	1				yes
Vitale et al. (13)	Vitale	32	32				
Vitale et al. (14)	Vitale	6		6			
Belkhir et al. (15)	Belkhir	1	1				yes
Kuijk et al. (16)	Kuijk	1	1				
Gattringer et al. (17)	Gattringer	2	2				
Roldan et al. (18)	Roldan	1	1				
Mitroulis et al. (19)	Mitroulis	1	1				
Calligaris et al. (20)	Calligaris	1	1				
Moser et al. (21)	Moser	1	1				yes
Hennig et al. (22)	Hennig	1	1				yes
Bilginer et al. (23)	Bilginer	1	1				yes
Petropoulou et al. (24)	Petropoulou	1	1				
Meinzer et al. (25)	Meinzer	1		1			
	Meinzer	5	5			12	
	Meinzer	1			1		
Ozen et al. (26)	Ozen	5	5			16	
Alpay et al. (27)	Alpay	1	1				yes
Stankovic Stojanovic et al. (28)	Stankovic	4	4			27	yes
Hacihamdioglu et al. (29)	Hacihamdioglu	1			1		
Estublier et al. (30)	Estublier	1	1				
Soriano et al. (31)	Soriano	1	1				
Ter Haar et al. (32)	Ter Haar	3	3				
Celebi et al. (33)	Celebi	1	1				yes
Mercan et al. (34)	Mercan	2	2				
Brik et al. (35)	Brik	7		7		9,5	
Başaran et al. (36)	Basaran	4	4			17	
	Basaran	4			4	12	
Ugurlu et al. (37)	Ugurlu (poster)	19			19		yes
Gül et al. (38)	Gül	9		9		22	
Cetin et al. (39)	Cetin	12	12			31	yes
	Cetin	8		8		18	yes
Eroglu et al. (40)	Eroglu	5	5			13	yes
	Eroglu	3		3		13	
	Eroglu	6			6	13	
Sevillano et al. (41)	Sevillano	1	1				yes
Alpa et al. (42)	Alpa	1			1		
Rossi-Semerano et al. (43)	Rossi	10	10			21	
	Rossi	1		1		21	
	Rossi	3			3	21	
Özçakar et al. (44)	Ozçakar	3		3		19	
	Ozçakar	10	10			14	yes
Sozeri et al. (45)	Sozeri	1		1			yes
Kucuksahin et al. (46)	Kucuksahin	24	24				
	Kucuksahin	2		2		29	yes
Laskari et al. (47)	Laskari	9		9		23	
	Laskari	5			5	43	
Ben-Zvi et al. (48)	Ben-Zvi	25	25				
Georgin-Lavialle et al. (49)	Georgin-Lavialle	1	1				
Abbara et al. (50)	Abbara	1	1				yes
Ozen et al. (51)	Ozen	20	20				
	Ozen	13		13			
Pecher et al. (52)	Pecher	13	13			31	
Barut et al. (53)	Barut	16	4	12			
Trabulus et al. (54)	Trabulus	9		9		33	yes
De Benedetti et al. (55)	De Benedetti	63		63			
Özçakar et al. (56)	Ozçakar	1	1				yes
	Ozçakar	4			4	23	yes
Jesenak et al. (57)	Jesenak	1			1		
Yildirim et al. (58)	Yildirim	3		3		57	yes
Yazilitaş et al. (59)	Yazilitas	11		11		14	yes
Ergezen et al. (60)	Ergezen (poster)	48	48				
Kohler et al. (61)	Köhler	29	29				yes
	Köhler	2			2		yes
Babaoglu et al. (62)	Babaoglu	15	15			34	
Gülez et al. (63)	Gülez	12		12		16, 5	yes
	Gülez	3			3	10	
Varan et al. (64)	Varan	33	33				yes
	Varan	11		11			yes
Varan et al. (65)	Varan	10	10				yes
	Varan	7			7	21	yes
Akar et al. (66)	Akar	133	133				yes
	Akar	19		19			yes
	Akar	18			18		yes
	Akar	2			2		yes
Berdeli et al. (67)	Berdeli	22		22		13, 8	
Hasbal et al. (68)	Hasbal	1	1				yes
Sargin et al. (69)	Sargin	14	14			41	
Eren Akarcan et al. (70)	Eren Arkacan	9		9		14, 3	
Kisla Ekinci et al. (71)	Kisla Ekinci	14	14			11	yes
Sendogan et al. (72)	Sendogan	4			4		yes
	**TOTAL**	**811**	**496**	**234**	**81**		

Anakinra was the main IL-1 inhibitor used (*n* = 496, 61.2%), rarely prescribed as an “on-demand” treatment (20/496, 4.0%). Two hundred and thirty-four (28.9%) patients were treated with canakinumab and 81(10.0%) with both IL-1 inhibitors, starting in all patients except 2 with anakinra before switching to canakinumab. The Supplementary Figure represents the prescription of IL-1 inhibitors in FMF since the first description in 2006.

We were unable to identify the patients who had been described more than once in the literature: first in case reports, than in case series and/or in retrospective studies. That is the reason why we were unable to assess bias or outcome assessments.

## Discussion

### Indications for IL1 Inhibition in FMF Patients

#### Colchicine Resistance

The main indication for the prescription of IL-1 inhibitors is colchicine resistance. However, the meaning of “colchicine resistance” has evolved over the past decade, and a consensus definition remains elusive. The criteria for insufficient response to colchicine are highly variable in the different studies. In many studies, no specific criteria are given to determine whether a patient is resistant or not; the indication to treat with IL-1 inhibition is often made only on the notion of “frequent attacks” despite colchicine treatment. Other studies give a more precise definition of colchicine resistance, but again the different definitions are highly variable. The highest agreement for colchicine resistance is the persistent elevation of acute phase reactants between the attacks (12, 15–20, 23, 24, 26–29, 33, 34, 36, 38–40, 44, 46, 49, 50, 52, 55, 57–59, 61, 63–65, 73). No consensus exists about attack frequency: some authors define colchicine resistance in patients who experience more than 1 typical inflammatory attack per 3 months (38, 53), while others refer to colchicine resistance if there are more than 2 typical attacks per trimester (46) and still others if the patient has monthly attacks (35, 40, 48, 51, 52, 55, 59, 62–65). None of the studies considers that attack frequency may vary with age and therefore the probable necessity to define this parameter differently in adults and children. Some studies take also into consideration the severity of inflammatory episodes in their definition of colchicine resistance (2, 4, 5, 7–11, 17–20, 27, 28, 34, 35, 38, 40, 44, 48, 49, 52, 53, 55, 57, 58, 73) but rarely give a precise definition for this item. Only very few studies include the notions of quality of life assessment or school or work attendance (4, 7–12, 18, 19, 27, 34, 53, 61, 73) into the definition of colchicine resistance, but without defining what is tolerable as absence from work or school or quality of life.

The maximum dosage of colchicine is usually set at 2 mg/day (27, 35, 38, 46, 54, 55, 66), but may change in the different studies, especially in children where doses vary with age in whom the standard dose is sometimes defined as 1.2 mg/m^2^/day (53). The standard and the most accepted minimal dose before considering resistance in the literature is the “maximum tolerated dose” (74).

The last factor of lack of precision is the difficulty to determine incompliant patients since there is no reliable and practical detection method to estimate active colchicine levels. Only one study had a standardized methodology to verify colchicine compliance by counting the remaining tablets (40). However, compliance with colchicine treatment appears to be low overall (10, 75, 76), which explains why the EULAR recommendations note that lack of compliance should be considered in all patients who do not respond adequately to colchicine (54).

#### Colchicine Intolerance/Toxicity

Therapeutic oral doses of colchicine in patients without hepatic or renal failure have few side effects and are generally well-tolerated. The most common side-effects are gastrointestinal, including diarrhea, vomiting and nausea. Gastrointestinal toxicity is dose dependent and may improve by diminishing lactose intake (77), or lowering the colchicine dose. Rarer acute adverse effects include myopathy, rhabdomyolysis, and myelosuppression (78). A colchicine neuromyopathy may occur with chronic daily use, particularly in patients whose dose has not been appropriately adjusted for renal disease (79–81). Of note fatalities during therapeutic use have been reported only in patients with chronic renal insufficiency taking unadjusted doses of colchicine or when colchicine has been given intravenously, or combined with CYP3A4 inhibitors (82–84). Due to all these side effects, the intolerance or toxicity of colchicine is therefore a possible indication for the treatment with IL-1 inhibitors.

The main reason for starting IL-1 antagonist in the different studies was the poor digestive tolerance of colchicine (12, 27, 46–48, 85), but in the various studies it was not specified whether means to increase digestive tolerance had been implemented before considering IL-1 inhibition. The second reason for IL-1 inhibition was neuromyopathy (15, 25, 31, 42, 47, 51, 58, 62, 68), occurring exclusively in patients with a risk factor such as renal disease or drug interactions. Myelotoxicity (24, 47) and hepatotoxicity (46, 58) was responsible for the initiation of an IL-1 inhibitor only on a very ad hoc basis. From these studies it appears that the intolerance or toxicity of colchicine is only exceptionally responsible for the need to initiate IL-1 blockade. This is particularly true in children who only exceptionally display co-morbidities that may decrease colchicine tolerance.

In view of the many aspects regarding colchicine treatment, it seems essential to extensively evaluate all these domains before considering colchicine resistance or intolerance. In Table 2, we propose a checklist to help the physician to assess the FMF patient before planning alternative or additional treatment plans to colchicine.

**Table 2:** Check list before considering colchicine resistance.

□	Verify that the diagnosis of FMF is accurate
□	Verify that reported symptoms are related to inflammation (Check inflammatory markers during symptoms)
□	Eliminate common causes of fever and pain (infection, leukemia, …)
□	Question the patient about personal, social or psychological problems that may be triggers for inflammatory attacks - Propose behavioral approaches for stress management
□	Ensure compliance at full dose for 3–6 months If the maximum dose is not reached, increase gradually the colchicine dosage by 0.5 mg (0.25 mg before the age of 5) every 3 months
□	Ensure colchicine tolerance by - Dietary modifications (limit lactose intake) - Splitting the total daily dose - Associating antidiarrheal and spasmolytic agents to colchicine
□	In patients with a sudden deterioration of FMF despite full compliance of colchicine, look for other causes of inflammation: - Inflammatory rheumatism, vasculitis - Mild myeloid hemopathy (in the elderly) - Chronic peritonitis or peritoneal mesothelioma (in the elderly)
□	Document prospectively the attack recurrence for 3–6 months in order to confirm the number of reported inflammatory episodes

#### Severe Complications or Associated Co-morbidities

##### Secondary amyloidosis

Amyloidosis is characterized by deposition of misfolded insoluble proteins in various organs and tissues. It is a life-threatening progressive disease unless underlying causes are treated early before irreversible organ damage occurs. AA Amyloidosis (AAA) is the most severe complication of FMF (86, 87). There is no cure for amyloidosis but it is preventable by suppression of inflammation. Hence, it is crucial to control inflammation in patient with preexisting AAA (88). As such, IL-1 inhibitors are good candidates in AAA FMF patients suffering from persistent inflammatory symptoms despite the regular use of colchicine at maximal dose.

At present, we were able to identify 160 patients who received IL-1 inhibitors for FMF and AA amyloidosis; they were mainly treated with anakinra compared to canakinumab, due to anakinra's short half-life. The exact number of FMF patients treated for AAA is not available because most series include FMF patients with and without AAA, and the details of data specific to patients with AA amyloidosis were not available, even in supplementary data.

When the data was available (*n* = 56), the foremost reasons for initiating an IL-1 inhibitor were in decreasing order of frequency: colchicine resistance (*n* = 24) (21, 22, 27, 28, 33, 39, 40, 44–46, 54, 56, 58, 72), AA amyloidosis and severe renal failure (*n* = 14) (12, 15, 41, 59, 71, 72), AA amyloidosis onset during the course of FMF (*n* = 13) (15, 23, 28, 31, 50, 72), colchicine intolerance (*n* = 7) (12, 15, 46, 58, 68, 72). Unfortunately, the exact reasons were not always specified in AAA patients from FMF series. Altogether, the main reason for IL-1 inhibitor prescription among patients with AAA secondary to FMF was severe kidney failure hindering the adjustment of the colchicine dosage necessary to normalize inflammatory markers. The second main reason was colchicine resistance defined by the persistence of raised inflammatory markers despite the regular daily intake of the maximum tolerated dose of colchicine, which was not always specified.

Anakinra, was mainly prescribed at 100 mg/day except for patients with end stage renal failure who received 100 mg of anakinra 3 times a week, each dialysis day. Canakinumab was mostly prescribed at 150 mg/month and was not chosen in case of end stage renal failure. Considering the recent prescription of IL-1 inhibitors in AAA secondary to FMF, there is not much hindsight to assess tolerance and efficacy in the long term but in the short term, the tolerance and efficacy seem correct. However, it is important to note that in the absence of renal failure, and if AAA reveals FMF, the treatment of choice remains colchicine (9). In case of AAA in FMF patients, colchicine should be tried first, starting at 1 or 1.5 mg/day and increased gradually in steps of 0.5 mg every 3 months during at least 6 months with a close monitoring of inflammatory markers and proteinuria, before starting IL-1 inhibitors. Indeed, in patients who are virgin of any colchicine treatment, the onset of AAA is first and foremost a sign of colchicine absence.

**Figure 1:**
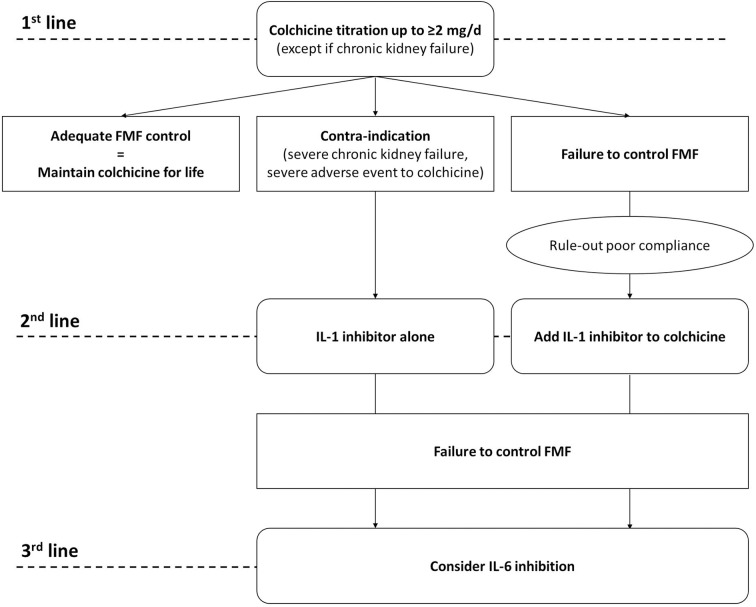
Proposal of a therapeutic algorithm for AA amyloidosis in patients with FMF.

Figure 1 proposes an algorithm for the decision to initiate an IL-1 inhibitor in FMF patients with AAA.

##### Associated co-morbidities

Unresponsiveness to colchicine—even if taken properly—may be due to associated inflammatory diseases. Most reports concern FMF associated ankylosing spondylitis with a good response to IL-1 inhibitors (29, 40, 46, 49, 63, 64) even if older studies showed also efficacy with TNF blocking agents (89, 90).

Other inflammatory conditions associated to FMF, such as Behçet's disease (23, 40), inflammatory bowel disease (27, 28, 33, 64), protracted febrile myositis (25, 30, 34, 40, 63), hidradenitis suppurativa (50) and Henloch-Schönlein purpura (25) also responded well to IL-1 inhibitors.

Finally, IL-1 inhibitors were described to be effective among FMF children with failure to thrive (36, 45) or children with an important need of corticosteroids (70); unfortunately the inflammatory status between attacks was not specified in these children.

In the light of these studies, patients with FMF previously well-controlled with colchicine alone, need a careful assessment for inflammatory comorbidities appearing later in the course of the disease. Indeed, the therapeutic implication of distinguishing this subgroup from the “true” colchicine resistance may be major: one can hypothesize that IL-1 inhibitors may be tapered and possibly withdrawn once the associated disease is controlled.

### Efficacy

To evaluate the efficacy of IL-1 inhibitors, phase 2 studies (in which the patient is his own control) and placebo-controlled studies provide the best information.

At our knowledge only one randomized, double-blind, placebo-controlled trial with anakinra in FMF patients who were colchicine resistant was published (48). In this study 25 patients were enrolled and randomized (12 in the treatment group and 13 in the placebo group). All the patients received maximum tolerated doses of colchicine during the whole study period. Seven patients discontinued the study, all of whom were from the placebo group, due to treatment failure or to side effects. Complete response was achieved in 7 patients of the treatment group whereas the 5 remaining patients had a partial response. For all the patients of the treatment group, anakinra improved furthermore life quality. In this specific study, anakinra combined to colchicine also decreased he number of joints attacks. These findings may support a role for dual therapy with colchicine especially in patients with FMF articular complications.

For canakinumab a first open-label pilot trial was conducted in 7 children who experienced at least 1 investigator-confirmed FMF attack per month (35). The median 28-day time-adjusted attack rate decreased from 2.7 to 0.3 in this study and the proportion of days that participants were experiencing an attack decreased from 24.2 to 3.6%. Furthermore, serum acute phase reactants levels normalized during the treatment period and health-related quality of life improved in all patients.

A second small open-label pilot trial with canakinumab in patients with monthly attacks, showed that monthly injections prevented attacks in 8/9 patients and reduced the frequency of attacks in the remaining patient (38). Furthermore, serum acute phase reactants levels (C-reactive protein and serum amyloid A protein) remained low throughout the treatment period in all the patients. Significant improvement was also observed in both physical and mental component scores.

These preliminary results in colchicine resistant FMF patients could be confirmed in a placebo-controlled phase 3 study with an injection every 4 weeks, in which 61% of 31 patients treated with canakinumab (150 mg or 2 mg/kg in children) vs. 6% of 32 patients in the placebo group had a complete response and did not experience any flare of the disease for 40 weeks while treated (55). This proportion increased to 71% of patients if the blinded dose in non-complete responders was increased to 300 mg (or 4 mg/kg in children) every 4 weeks. A dosing interval of every 8 weeks was enough to maintain complete disease control in 46% of patients with colchicine resistant FMF. In patients who did not have a complete control of the disease, the mean attack frequency decreased from more than 30 to <2 per year and remaining attacks seemed less severe.

Overall these studies provided evidence of the pathogenic role of IL-1 in colchicine-resistant familial Mediterranean fever. They also showed that IL-1 inhibition is an efficacious option for controlling and preventing flares –at least at the short term- in these patients.

### Anakinra vs. Canakinumab

Although canakinumab is the only drug approved in Europe for the treatment of colchicine resistant FMF, in literature, experience with anakinra is also significant. Up to now, there are no comparative effectiveness assessment studies for canakinumab vs. anakinra. The preference of one treatment over the other can therefore be based only on indirect data. Significant reasons to prescribe anakinra rather than canakinumab are the price and/or reimbursement conditions of the drugs, explaining probably partially why the experience in the literature with anakinra is more substantial than with canakinumab. By analyzing studies in which patients have switched from one treatment to another, it seems that the main reason for switching is the ease of administration of canakinumab and/or the loss of compliance to anakinra after longer periods of use (36, 43, 56, 61, 64, 66, 72). A second reason for switching from anakinra to canakinumab is the occurrence of injection site reactions or other side effects with anakinra (such as urticaria or the rise of liver enzymes) which seem less prominent when taking canakinumab (40, 42, 43).

Interestingly the loss of efficacy of the first line IL-1 inhibitor can also be a reason for switching from one IL-1 inhibitor to the other. An inadequate or partial response has principally be described with anakinra (mainly after an initial good response and a secondary recurrence of symptoms), followed by a better response with canakinumab (29, 36, 40, 43, 47, 57, 64, 66, 72). However, in none of these reports the adherence to daily injections has been assessed. Moreover, that the worsening of the clinical picture with IL-1 antagonist could be secondary to other potential non-FMF conditions was only exceptionally discussed. An inadequate response with canakinumab has also been described in 2 reports (36, 66); both reports relate that canakinumab treatment was changed to anakinra for clinical and/or laboratory worsening, with a good response. These observations raise the question of whether the efficacy of IL-1 inhibitors is sustainable over the long term.

At present and in the absence of comparative studies both treatments seem to be an equal option for the management of colchicine resistant or intolerant FMF patients.

### Maintenance Therapy vs. on Demand Treatment

The particularity of FMF is that the disease evolves by flare-ups. Usually, the only treatment offered are NSAIDs (naproxen, diclofenac, indomethacin, etc) that may alleviate symptoms during attacks but which are rarely completely effective (91). It therefore seems quite logical to offer intermittent treatment with IL-1 inhibitors to patients who continue to have attacks despite proper colchicine treatment. Paradoxically, this attitude has been little investigated. We identified simply 3 publications, concerning 20 patients treated with anakinra only during flares of the disease (19, 43, 92). The most interesting data is described in a retrospective study of Babaoglu and co-authors who investigated retrospectively The Gazi FMF cohort (92). The cohort is made up of 689 FMF patients of whom 78 patients were treated with IL-1 inhibitors among those 15 were treated with an on-demand anakinra protocol. Patient reporting attack severity, duration, frequency, absenteeism were significantly improved when receiving an on demand treatment with anakinra. Furthermore, prophylactic on-demand use of anakinra in patients with prominent triggers seemed also successful. All the patients continued the maximum tolerated dosage of colchicine and none had persistent inflammation before starting the on-demand protocol. The authors conclude that the use of anakinra during the prodromal period would be a reasonable approach for halting or alleviating symptoms of an impending attack allowing patients to diminish the loss of workdays and to improve the quality of life. Another advantage of this approach would be reducing cost and adverse effects of continued use of IL-1 inhibitors in selected patients with marked prodromes or triggers and low risk of amyloidosis.

### Safety

In the different studies, the safety of LL-1 inhibition seemed generally good, at least at the short term. Only one case series reported a death following a treatment with canakinumab (59). The patient had end stage renal disease and severe multiorgan amyloidosis and died due to sepsis and peritonitis 1 year after cessation of IL-1 inhibition treatment. One opportunistic infection (fungal pneumonia) was reported in one patient receiving canakinumab (66). No malignancies were reported in any of the studies or case series.

**Figure 2:**
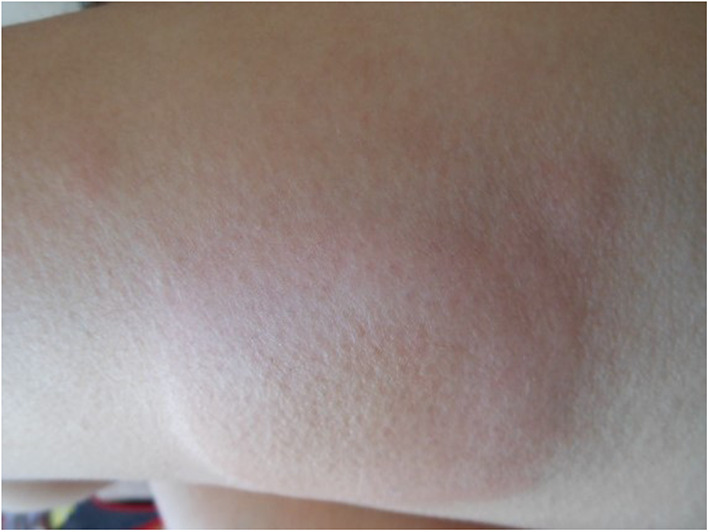
Example of an injection site reaction with anakinra.

The main reported side effect were local injection site reactions (17, 20, 25, 36, 40, 42, 43, 46, 48, 55, 57, 66, 69). Nevertheless, this side effect seems far more frequent in patients treated with anakinra than with canakinumab. Figure 2 shows an illustrative example of such a local site reaction. Up to now severe (43, 60, 66) or mild anaphylactic reactions (16, 40) were described only with anakinra.

The second most reported side effects were infectious complications, in a probably equivalent manner regardless of the type of IL-1 inhibitor used. The infections concerned chiefly the upper and lower respiratory tract (28, 35, 38, 48, 55) and were sometimes considered to be severe (12, 17, 39, 40, 56, 59, 66). Cutaneous infections (55, 63, 66) or viral infections of the herpes simplex group (54, 66) were also reported.

Anakinra seems to be more often responsible for cases of leucopenia (28, 37, 40, 62, 66), whereas headache (considered occasionally to be severe) seems to be observed more frequently with canakinumab (25, 38, 48, 55, 57).

Overall the safety profile of IL-1 inhibitors seems not different in FMF patients than in the other diseases including cryopyrin associated periodic syndrome, rheumatoid arthritis, adult-onset Still's disease and systemic-onset juvenile idiopathic arthritis. However, longer studies on FMF and post-marketing real-life experience are needed to verify the persistence of the relatively good IL-1 inhibitor tolerance on the long term.

## Conclusion

The results from the present review suggest that IL-1 inhibitors are good candidates for colchicine resistant and/or intolerant FMF patients. IL-1 inhibitors have the potential to improve patient outcome even in patients with co-morbidities or severe complications in whom inflammation control is difficult to achieve with colchicine alone. Nevertheless, current data are limited and further evaluation of long-term efficacy and safety of IL-1 inhibitors are necessary, in order to provide robust evidence in this domain.

## Author Contributions

VH conceived and wrote the review. CV and AF performed the literature search. VH and SG-L approved and validated the selected articles and checked the data assessment. All authors agreed to the final version of the manuscript.

## Conflict of Interest

VH and SG-L have received counseling fees or for travel expenses from Novartis and SOBI, manufacturer of canakinumab and anakinra, respectively, but not for this work. The remaining authors declare that the research was conducted in the absence of any commercial or financial relationships that could be construed as a potential conflict of interest.

## Abbreviations

AAA: amyloid type A protein amyloidosis formerly known as secondary amyloidosis
FMF: familial Mediterranean Fever
IL-1: interleukine 1
*MEFV* gene: mediterranean fever gene
NSAIDs: non-steroidal anti-inflammatory drugs
TNF: tumor necrosis factor.
