# Supplementary material for: The Use of Interleukine-1 Inhibitors in Familial Mediterranean Fever Patients: A Narrative Review
Source: Front Immunol. 2020 May 28;11:971. doi: 10.3389/fimmu.2020.00971 (PMC7326122; doi:10.3389/fimmu.2020.00971)

Supplementary figure: Cumulative number of FMF patients treated with Il-1 inhibitors and reported in the literature since their first use in 2006.


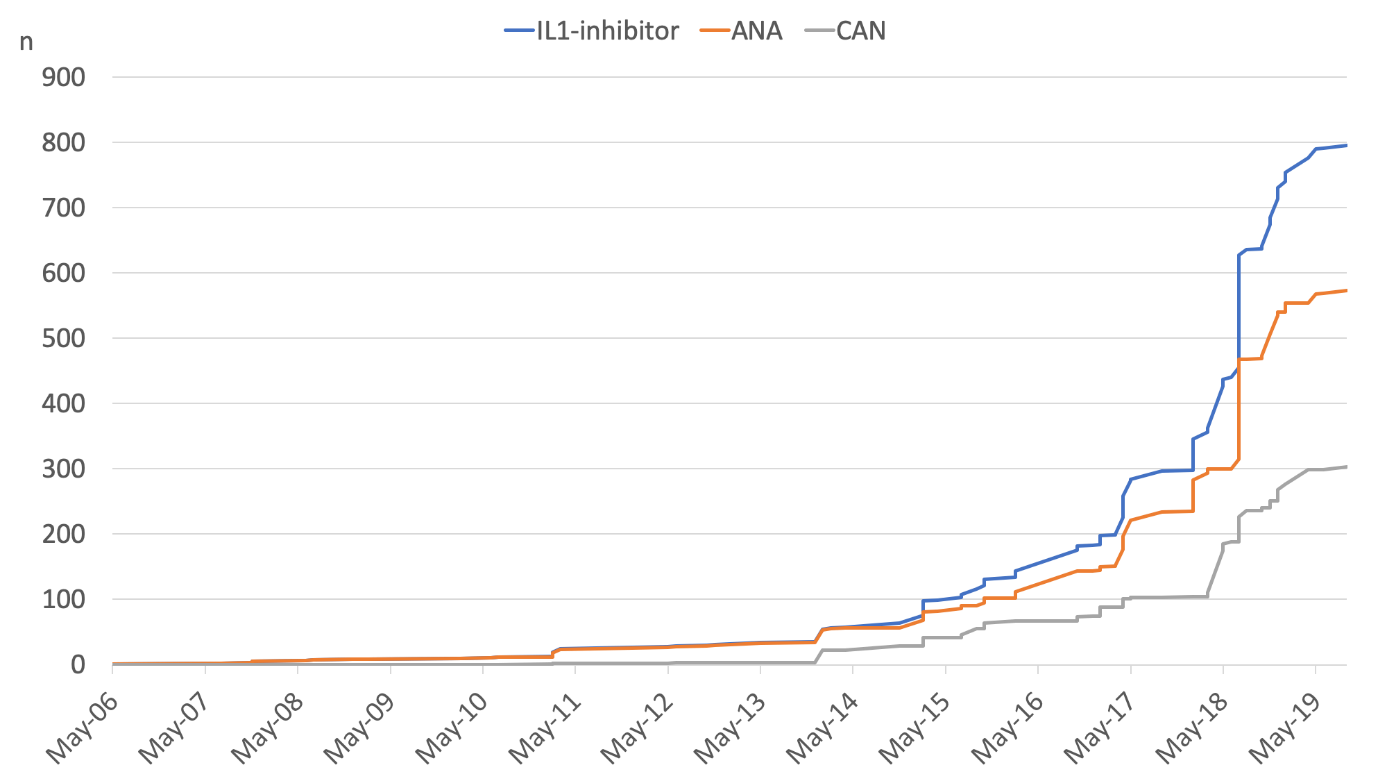

Supplement: Supplementary file 1 [file Table_1.DOCX]
